# Communicating genetic test results within the family: Is it lost in translation? A survey of relatives in the randomized six-step study

**DOI:** 10.1007/s10689-016-9889-1

**Published:** 2016-02-20

**Authors:** Mary B. Daly, Susan Montgomery, Ruth Bingler, Karen Ruth

**Affiliations:** 1 Department of Clinical Genetics, Timothy R. Talbot Jr. Chair for Cancer Research, Fox Chase Cancer Center, 333 Cottman Avenue, Philadelphia, PA 19111 USA; 2 Risk Assessment Program, Department of Clinical Genetics, Fox Chase Cancer Center, 333 Cottman Avenue, Philadelphia, PA 19111 USA; 3 Department of Clinical Genetics, Fox Chase Cancer Center, 333 Cottman Avenue, Philadelphia, PA 19111 USA; 4 Department of Biostatistics, Fox Chase Cancer Center, 333 Cottman Avenue, Philadelphia, PA 19111 USA

**Keywords:** Genetic testing, BRCA1/2, Cancer risk, Family communication

## Abstract

Genetic testing for cancer susceptibility genes is increasingly being integrated into medical care. Test results help inform risks of the individual being tested as well as family members who could benefit from knowing the results. The responsibility for informing relatives of genetic test results falls on the proband, the first family member being tested. However, there are several challenges associated with sharing genetic test results within families including incomplete understanding of test results, emotional distance among family members, and poor communication skills. In this paper we describe the communication process between probands randomized to receive *BRCA1/2* genetic test results in an enhanced versus a standard of care counseling session, and their first degree relatives with whom they shared results. We contacted 561 first degree relatives of probands who had undergone *BRCA1/2* genetic testing to measure their level of understanding of the test results, their difficulty and distress upon hearing the results, the impact of the test results on their risk perception, and their intention to pursue genetic counseling/testing. 82.1 % of relatives correctly reported the test results of their proband. Distress upon hearing the test result was highest for those relatives whose proband received informative test results. Relatives reported a decrease in cancer risk perception after hearing the test results, regardless of the type of result. Intention to pursue counseling/testing was low, even among those relatives whose proband received informative test results. Male relatives were less likely to be informed of test results and more likely to forget hearing them. These results suggest ways to improve the communication process within families.

## Introduction

Genetic testing for cancer susceptibility genes is increasingly being integrated into medical care. There is growing interest in the psychosocial impact of genetic testing, both on the individual who has been tested and on family members who could benefit from knowing the test results. Sharing genetic test results (GTRs) with relatives may help them to clarify their own risk of cancer, and to identify optimal risk management strategies. The responsibility for informing relatives of genetic test results falls on the proband, the first family member being tested [1]. Accurate transmission of genetic risk information depends on a basic understanding of genetic principles and a certain level of comfort with numbers and risk estimates. There are several challenges associated with sharing genetic test results within families. Possible test results include true positive, true negative (informative results), indeterminate and inconclusive (uninformative results) (Table 1). Both true positive (deleterious mutation found in the proband) and true negative (there is a deleterious mutation in the family, but the proband was not found to carry it) are considered informative test results for the first degree relatives, as both indicate an increased risk for the relative to carry the same mutation. Inconclusive and indeterminate results are associated with uncertainty and are conveyed to relatives less frequently than conclusive results [2–4]. Communicating genetic test results is more distressing for women who are carriers of deleterious *BRCA1/2* gene mutations, who are the first tested among their siblings, and among those whose siblings prove to be non-carriers [5]. Mutation carriers have reported difficulty communicating test results [6, 7] and guilt about potentially having transmitted a mutation to their children [8]. Cancer-related emotional distress is also a barrier to diffusion of test results [9]. While there is limited data about the reaction of relatives with whom genetic test results are shared, there is evidence that open, positive family relationships increase the likelihood of disclosure of test results, while emotional distance, family conflict, and loss of contact decrease the likelihood of disclosure [10–12].

**Table 1:** Possible genetic test results

Result	Description
True positive	Mutation was identified in the proband that could increase cancer risk
True negative	Mutation was identified in the family, but was not inherited by the proband
Inconclusive	Alteration of uncertain significance was found in the proband
Indeterminate	BRCA alteration was not identified in proband or any other family member

The Six Step Communication Study was designed to provide communication skills to probands for transmitting their genetic test results to their at-risk adult family members. As part of this study, after the disclosure of test results to the proband, those relatives for whom the proband had given permission were surveyed by phone regarding factors associated with the communication process, and their ability to understand and cope with the information provided to them by the proband. The purpose of this component of the study was to explore the accuracy of the relatives’ understanding of GTRs, the implications for their own risk, and their level of distress associated with the information they received. We were also interested in those variables associated with level of accuracy of test results, including relative age, gender and relationship to the proband. In this paper we describe the communication process between probands undergoing genetic testing for *BRCA1/2* and their first degree relatives with whom they shared results.

## Methods

The details of the Six Step Communication Study have been published previously [13]. Women undergoing genetic counseling and testing for *BRCA1/2* who reported having adult first degree relatives with whom they planned to share test results were eligible. The probands were randomized to a communication skills-building intervention or a wellness control session in conjunction with genetic counseling. The two study arms were stratified by breast/ovary cancer status (affected vs unaffected). At pre-test counseling, probands were asked to provide permission to contact at least one adult first degree relative (FDR) with whom they planned to share results. Those relatives were to be contacted by phone approximately 3 months after their proband received test results for a short survey following disclosure of results. Probands completed a relative identification form with name, address, and phone number of FDRs that would be contacted. A phone log was generated for each relative.

At the time of the phone call, a verbal consent to participate in the survey was obtained. The survey consisted of 18 questions and took about 10–15 min to complete. The survey questions included an interpretation of the test result of the proband, the relative’s cognitive and emotional response to the genetic information, the relatives’ self-perceived risk before and after hearing the genetic information, and the relative’s intention to pursue genetic counseling and genetic testing. Included in the survey were open-ended questions probing reasons for intended actions.

### Statistical analysis

We used Pearson’s Chi square tests to assess differences in characteristic categorical variables and outcomes including the proportion of relatives given “permission-to-contact” by the proband, the proportion who participated in the phone survey, the proportion who reported that the proband shared GTRs, and the proportion who gave a correct interpretation of the GTR. For characteristics with more than two levels, (e.g. generation with levels of parent, sibling, child), pairwise comparisons were made if the overall Chi square test was statistically significant. Continuous variables were compared with *t* tests. Changes in perceived risk perception were compared with McNemar’s test. Statistical tests were 2-sided with 5 % Type I error. Analyses were performed using SAS^®^ statistical software, version 9.3 (Cary NC).

## Results

In a previous publication we reported that overall, probands reported sharing their test result with 80 % of 838 eligible FDRs, which is consistent with the literature. The majority of probands shared test results with 1 or 2 eligible relatives. Probands were more likely to report sharing test results with female relatives, and with their adult children than other members of the family. There was no difference in the percentage of probands who shared their test results, or their level of distress with the communication process between the two study arms [14]. This study focuses on the reactions of the relatives with whom probands reported sharing their test results.

**Fig. 1:**
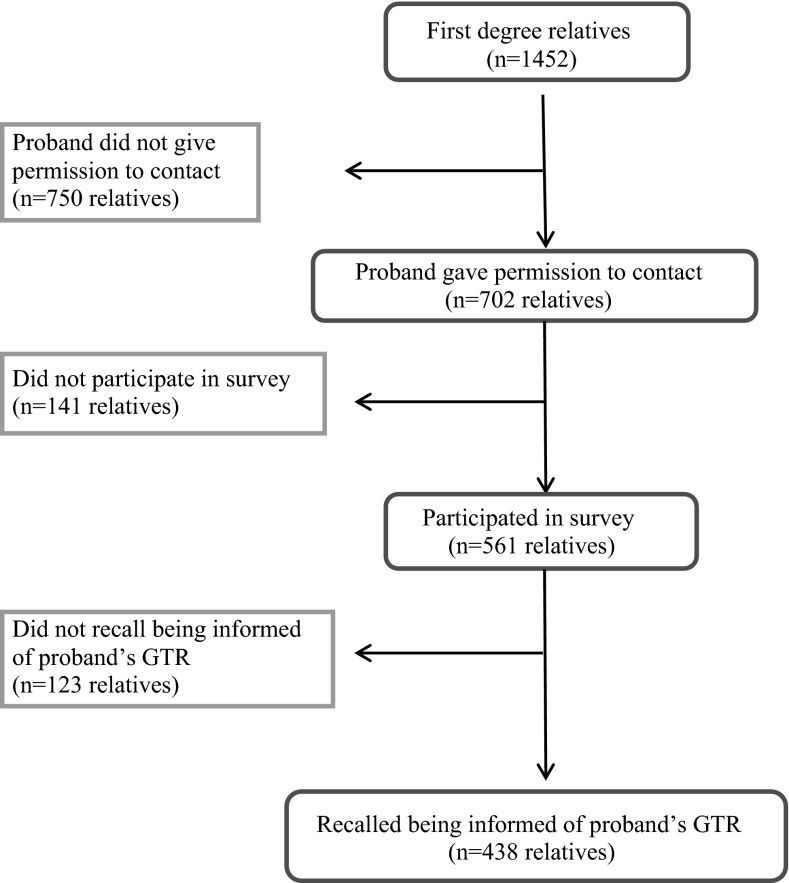
Schema showing identification of relatives included in these analyses

A total of 1452 living adult first degree relatives were identified by the study team from family history data originally provided by the 345 probands in the study (See Fig. 1: Schema). Probands provided permission for the study team to contact 702 (48.3 %) of these relatives. Probands were more likely to give permission to the study team to contact their female relatives than their male relatives (Table 2). Permission to contact did not differ significantly by the relatives’ relationship to the proband or the study arm of the intervention.

**Table 2:** Association of relative characteristics with proband’s permission to contact

Characteristic	N relatives	Permission given to contact		Permission not given to contact		*p* value
		n	Row %	n	Row %	
All	1452	702	48.3	750	51.7	
Relative’s gender						
Female	770	437	56.8	333	43.2	<0.0001
Male	682	265	38.9	417	61.1	
Relative’s generation (to proband)						
Parent	233	111	47.6	122	52.4	0.17
Sibling	781	363	46.5	418	53.5	
Child	438	228	52.1	210	47.9	
Proband study arm						
Control	667	327	49.0	340	51.0	0.065
Intervention	785	423	53.9	362	46.1	

We were able to contact 561 (80 %) of the 702 relatives. The other 141 relatives either were not able to be contacted, or upon initial contact were uninterested in participating in the survey. Those relatives who did not participate in the survey did not differ by age, gender, relationship to proband or type of test result from those who did participate (Table 3). One-hundred twenty-three (22 %) of the relatives who were contacted reported that the proband had in fact not shared test results with them, despite the proband having reported that they had communicated their test results. Reporting of sharing GTRs differed by relative characteristics (Table 4). Female relatives were more likely to report receiving the test information from the proband than males relatives, as were adult children of the proband. Also relatives whose proband’s test results were informative were more likely to report that they did receive the test results than those that were uninformative. We did not pursue the remainder of the survey with those relatives who indicated they had not been told the test results. We continue here with the 438 relatives who did report hearing the test results.

**Table 3:** Relative characteristics by participation in phone survey

	Participating (N = 561)		Not participating (N = 141)		*p* value
	n	Column %	n	Column %	
Relative’s relationship (to proband)					
Mother	49	8.7	11	7.8	0.70
Father	39	7.0	12	8.5	
Sister	187	33.3	38	27.0	
Brother	107	19.1	31	22.0	
Daughter	121	21.6	31	22.0	
Son	58	10.3	18	12.8	
Relative’s gender					
Female	357	63.6	80	56.7	0.13
Male	204	36.4	61	43.3	
Relative’s generation (to proband)					
Parent	88	15.7	23	16.3	0.75
Sibling	294	52.4	69	48.9	
Child	179	31.9	49	34.8	
In relatives, proband GTR distribution					
Informative	80	14.3	27	19.2	0.15
Non-informative	481	85.7	114	80.9

**Table 4:** Characteristics associated with reported sharing of GTR by relative

	Relatives participating	Relative reported proband shared GTR		Relative reported proband did not share GTR		*p* value
	N	n	Row %	n	Row %	
All	561	438	78.1	123	21.9	
Relative’s relationship (to proband)						
Mother	49	41	83.7	8	16.3	<0.0001
Father	39	29	74.4	10	25.6	
Sister	187	149	79.7	38	20.3	
Brother	107	63	58.9	44	41.1	
Daughter	121	113	93.4	8	6.6	
Son	58	43	74.1	15	25.9	
Relative’s gender						
Female	357	303	84.9	54	15.1	<0.0001
Male	204	135	66.2	69	33.8	
Relative’s generation (to proband)						
Parent	88	70	79.6	18	20.4	<0.0001
Sibling	294	212	72.1	82	27.9	
Child	179	156	87.2	23	12.8	
Informative GTR						
Informative	80	72	90.0	8	10	0.0054
Non-informative	481	366	76.1	115	23.9	
Study arm proband						
Control	273	204	74.7	69	25.3	0.062
Intervention	288	234	81.3	54	18.7	

Relatives were asked about their interpretation of the test results. We compared their responses to the actual test results obtained from the medical record to determine the accuracy of the relatives’ interpretation. Overall, 82 % of the relatives’ interpretation of the test result was concordant with the true result. Relatives were significantly more likely to correctly report informative test results than uninformative results (91 vs. 80 %, *p* = 0.029). The correct interpretation of the test result did not differ by age of the relative, gender, relationship to the proband, or study arm of the proband. (Table 5) Of interest, 10.5 % reported that they were told the test result but were not able to remember it. Those relatives reporting that they did not remember the test result were significantly more likely to be male gender than female gender (57 vs. 43 %, *p* > 0.001). (Data not shown).

**Table 5:** Characteristics associated with correct interpretation of GTR results

	N	Correct interpretation of GTR		Incorrect interpretation of GTR		*p* value
		n	Row %	n	Row %	
All	392	322	82.1	70	17.9	
Relationship to proband						0.29
Mother	35	26	74.3	9	25.7	
Father	23	17	73.9	6	26.1	
Sister	140	120	85.7	20	14.3	
Brother	52	39	75.0	13	25.0	
Daughter	108	93	86.1	15	13.9	
Son	34	27	79.4	7	20.6	
Gender						0.054
Female	283	239	84.5	44	15.5	
Male	109	83	76.2	26	23.8	
Generation						0.21
Parent	58	43	74.1	15	25.9	
Sibling	192	159	82.8	33	17.2	
Child	142	120	84.5	22	15.5	
Age, years						
Correct interp.	n = 321	Mean (SD) = 45.6 (16.2)				0.47
Incorrect	n = 70	Mean (SD) = 47.2 (18.7)				
Informative GTR						0.029
Informative	69	63	91.3	6	8.7	
Non-informative	323	259	80.2	64	19.8	
Study group of proband						0.38
Control	181	152	84.0	29	16.0	
Intervention	211	170	80.6	41	19.4	

Overall, 14 % of the relatives found the test information very or somewhat difficult to understand. Difficulty in understanding did not differ by age, gender, relationship to proband, test result, or study arm of the proband. Thirteen percent found the information very or somewhat upsetting. Only type of test result was significantly related to reporting distress. Relatives of probands whose test results were informative were significantly more likely to find the results upsetting (35 vs. 8 %, *p* < 0.001).

**Fig. 2:**
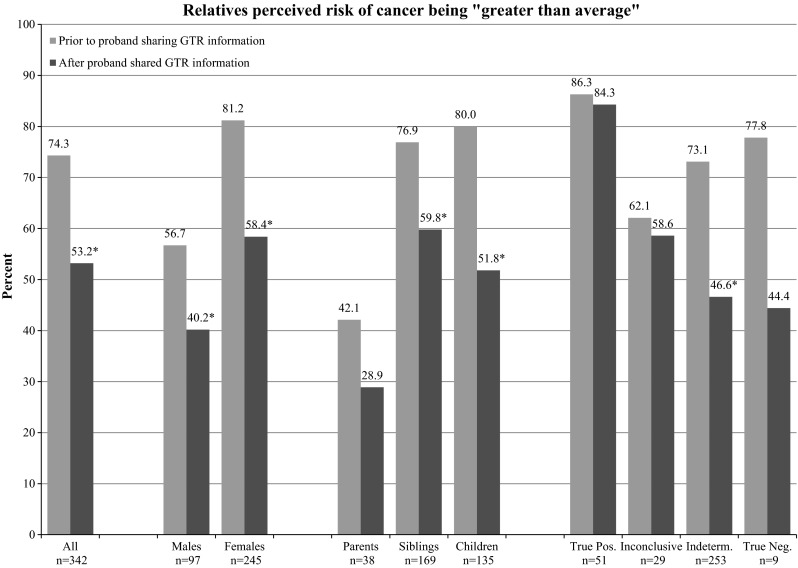
Relatives perceived risk of cancer being “greater than average”, before and after proband shared GTR (*p ≤ 0.001)

Relatives were asked to report what they believed their own risk for developing cancer was before receiving the proband’s test results, and if that changed after hearing the test results. Seventy-four percent of relatives reported that they had believed their risk for cancer was greater than the average before hearing the test results. After hearing the test results, this percentage dropped to 53 %. The drop in perceived risk was significant for within genders, within generations, and for indeterminate test results. Unexpectedly, perceived risk dropped even among relatives with informative test results, although they did not reach statistical significance due to small numbers (Fig. 2).

Of relatives without prior genetic counseling or genetic testing, 31.5 % reported intention to pursue genetic counseling (Table 6). Intention was highest for those relatives who reported that their proband had an informative test result, both positive and true negative. The adult children of the proband reported significantly higher intention to pursue genetic counseling than the siblings or the parents. Similarly, 35 % of the relatives reported intention to pursue genetic testing. Again, intention was highest for those relatives who reported that their proband had an informative test result. As seen with counseling, the adult children of probands reported significantly higher intention to pursue genetic testing than the parents or the siblings. Intention to pursue counseling or testing did not vary by study arm of the proband, age, gender, accuracy of the relative in reporting the proband’s test result, or difficulty in understanding the test result or distress in hearing the test results. The reasons most cited for intending to pursue genetic counseling were to “find out my risk” and to “find out about my children’s risk.” The reasons most cited for intending to pursue genetic testing were to “find out if I carry an altered gene” or to “find out about my children’s risk.” In neither circumstance were concerns about insurance or discrimination cited as significant reasons for not intending to pursue counseling or testing.

**Table 6:** Factors associated with intention to pursue GT, GC, either (n = 324 after excluding those with prior GC or GT)

	N	Intend to pursue GC			Intend to pursue GT		
		n	Percent	*p* value	n	Percent	*p* value
All							
Without prior GC/GT	324	102	31.5		114	35.2	
Study group							
Control	149	52	34.9	0.22	56	37.6	0.40
Intervention	175	50	28.6		58	33.1	
Proband GT result, groups							
Informative	47	24	51.1	0.0018	23	48.9	0.033
Non-informative	277	78	28.2		91	32.9	
Interpretation of GTR							
Correct	260	82	31.5	0.96	92	35.4	0.88
Incorrect	64	20	31.3		22	34.4	
Gender of relative							
Female	221	74	33.5	0.26	78	35.3	0.95
Male	103	28	27.2		36	31.6	
Generation of relative							
Parent	50	9	18	0.021	8	16	0.0002
Sibling	157	47	29.9		50	31.9	
Child	117	46	39.3		56	47.9	
Relative had difficulty in understanding GTR							
No	279	87	31.2	0.70	98	35.1	0.87
Yes	44	19	34.1		16	36.4	
(n = 1 msng excluded)							
Relative found GTR upsetting							
No	279	82	29.4	0.021	97	34.8	0.52
Yes	40	19	47.5		16	40.0	

## Discussion

Intrafamilial communication of health threats is a complex and dynamic process. Our data illustrates some of the limitations of relying on the proband to be the primary conduit of genetic information to their family members. In our current health care model, the decision to share test results resides with the proband, leaving some relatives without access to the information. While probands indicated an intention to share their test results with the majority of their first degree relatives, not all relatives were informed by the proband. Failure to share test results may be a function of poor communication within the family, or emotional distance from some relatives. When deciding with which relatives to share test results, probands may weigh such factors as the perceived vulnerability or resilience of the relative, their level of maturity, their coping skills and their stage of life [15]. Some probands may be confused about which relatives are at risk for inheriting a breast cancer related mutation. Probands may fear negative consequences such as causing distress or anxiety, or having an adverse impact on their relationship [16–18]. Some probands may rely on other family members to share or disseminate the information within the family, e.g., relying on sisters to tell brothers [19]. In addition to variability in terms of with whom genetic test results are shared, probands also vary in how much information to disclose, and when they plan to share the information [20]. Another indication of the selective nature of the communication process is the finding that although probands reported sharing test results with 80 % of first degree relatives, they declined permission for the study team to contact over 50 % of those eligible relatives. Permission to contact was significantly less for male relatives. Probands may withhold permission to contact certain relatives with whom they have not completed the process of informing, those with whom they lacked confidence about their ability to fully explain the meaning of the test results, or those for whom they felt reluctant to involve in a research study.

Over 80 % of the relatives participating in the survey with whom test results were shared correctly reported the proband’s test result. However our data suggests that, although the majority of the relatives surveyed were able to report the correct definition of the test result, the correct interpretation and relevance of the results for their own cancer risk appears to have eluded many of them. First, over 20 % of the relatives with whom probands reported sharing results denied even hearing their proband’s test result, suggesting that these relatives did not grasp the significance of the information, or did not consider it relevant to their own cancer risk. Eighteen percent of the relatives reported hearing the test results but were not able to correctly identify it. Interpretation of test results was most discordant for non-informative test results, either indeterminate results or variants of uncertain significance, which carry a degree of uncertainty as to their interpretation. The discordance we observed is likely due to some combination of a selective disclosure, or filtering of the information on the part of the proband [15], a lack of understanding of the test result on the part of the proband and/or the relative, a failure to appreciate the relevance of the test result by the proband and/or the relative, or patterns of denial or blunting on the part of the relative [19]. That this discordance was more common among relatives for whom the results were non-informative is consistent with these scenarios. Gaps in the quality of communication are also supported by the finding that over 10 % of the relatives who reported receiving the information from the proband actually forgot the nature of the test result. This again speaks to the lack of perceived relevance on the part of some relatives.

The importance of accurate risk perception is thought to be a significant motivator of positive health behaviors [21], although not all studies demonstrate this connection. Specifically, the receipt of information about a cancer susceptibility gene mutation has not always been found to alter risk perception [22]. In our data, the relatives reported a decrease in their personal perceived risk after hearing the proband’s test results, even among those whose proband received informative test results. Keeping in mind that all of these relatives have a family member with a cancer risk high enough to warrant genetic testing, this downward trend in perceived risk suggests that there is limited understanding of the significance of the family history, and of the meaning of the test result among many of the relatives. The receipt of uninformative test results is likely to be problematic for both the proband and her relatives, resulting in uncertainty about the true risk for cancer in the family. Uninformative test results however do not eliminate risk as there may be other genetic and/or environmental factors explaining the strong family history. There is a large body of literature indicating that risk perception is more than a numerical estimate, but rather reflects a complex psychosocial process involving life experience with a health threat such as cancer, personality and emotional state [3, 21]. Risk information may threaten one’s sense of control over life and impact feelings of personal vulnerability.

Similar to other studies [7, 12, 19], less than one third of relatives surveyed intended to pursue genetic counseling/testing, even though in families with inconclusive or indeterminate test results, testing of further family members may help to clarify the genetic risk. While intention to pursue genetic counseling/testing was highest for those relatives whose probands had an informative test result, almost 50 % of that group did not report intention to pursue further evaluation for their own risk, although they had a 50 % chance of carrying the familial mutation. This suggests that although the majority of relatives could accurately classify the test result, it is not clear if they understood its meaning for their own risk. Relatives who are not familiar with the process of genetic testing and counseling, or are unaware of the implications of the genetic test for their own risk are not likely to make informed decisions about how they may benefit from counseling/testing.

Our data also confirms the finding in our previous work and that of others of a pronounced gender difference in the receipt and understanding of genetic information within the family [14, 16, 17, 21]. The men in the family were less likely to be chosen by the proband for contacting to participate in the study. Among those men for whom we received permission to contact, they were more likely to deny hearing the test results or to forget the test results. In most western cultures, women are typically assigned the role of health maintenance within the family. The culturally accepted gender roles within the family have a strong impact on the ways men and women understand and respond to health threats. The tendency for avoidance of health threats or denial of their personal relevance among men may explain the greater number of male relatives who reported not hearing or having forgotten the test results.

These findings indicate that sharing of genetic test results by probands with their adult first degree relatives is variable, that the information shared may not be well understood and that relying on the probands to share their test results with their relatives is fraught with limitations which may compromise the value of the information for the relatives. In the setting of hereditary cancer risk, the nuances of family communication, including family cohesion, family support and communication styles will impact the quality of the information shared. Genetic risk information transmitted from the proband to relatives can be subject to misinterpretation, inaccuracy, or filtering on the part of the proband. Even when the proband accurately reports the test result, relatives, who have not had the benefit of genetic counseling may misunderstand the meaning of the test or its relevance to them.

### Strengths

Two aspects of our study design lend weight to our findings. This study is one of the few studies that does not rely on the proband’s report of sharing test results, but obtains the information directly from the relatives. The inclusion of men in the study adds important information to the differences in gender roles observed in other studies of sharing genetic information.

### Weaknesses

A potential weakness is the inability to survey those relatives for whom the proband did not give permission to contact (52 %). While this may introduce a selection bias, we were constrained by our ethical obligation to honor the wishes of the proband. Our reliance on memory for the relative’s risk perception prior to hearing the proband’s test result introduces the potential for recall bias. Our relative survey did not include a measure of familial cohesion, family communication patterns, or level of familial social support, all of which have been found to predict the frequency of communication of genetic test results to family members [11, 23]. Because the sample was primarily Caucasian we were not able to measure differences in communication patterns by ethnicity. We do not have any data on the actual nature of the communication with relatives, what topics were discussed and if the conversation differed by which relative was told. Relatives’ report of genetic test result was taken at a snapshot in time, whereas discussion of genetic risk within a family can be a long-term evolving process.

## Future directions

This data has significant implications for the genetic counseling process. We have identified gaps in the process of communicating genetic information within families which can seriously compromise the value of the genetic risk information for family members. We identified both failure to share results with certain relatives, as well as evidence of selective sharing of results. This evidence includes failure to remember hearing the test results, hearing the test results but forgetting what they were, and hearing the test results but incorrectly reporting them. These gaps in communication can have significant adverse effects for relatives, who may fail to become aware of their own level of risk, who may as a result not take advantage of potential risk reducing options, and who may serves as a barrier for the further communication of information within their own nuclear family. This impact is supported by the relatively low uptake of genetic risk counseling and/or testing among the informed relatives. That these gaps differ by both the nature of the test result and by personal and demographic characteristics attests to the complex nature of the communication process which is subject to many factors. These findings have significant implications for policy regarding optimal counseling models, particularly in view of the recent clinical introduction of multi-gene panels and nest generation sequencing to determine genetic risk. Although limited, we have enough data to propose that by being more cognizant of the burden placed on probands to share their genetic test results, genetic professionals can explore with the proband the nature of the family dynamics, their communication histories with relatives and the anticipated reactions of family members. They can alert the proband to the potential impact of the genetic risk information on family dynamics and provide guidance on communication strategies. They can emphasize the relevance of test results to male members of the family. Given the universal finding that male relatives are less likely to be informed of genetic information within the family, educational materials that are specifically designed to meet the information needs of men may also improve the communication process within the family. Finally, they might also integrate long term follow up of the proband and their families into the counseling process to help the family navigate the process of incorporating the genetic information into the family identity [24]. This study illustrates a critical need within the genetic community for more in-depth research on the actual content of genetic information shared, and the contribution of family dynamics and patterns of familial communication of genetic information in diverse ethnic populations.
